# Spatio-temporal categorization for first-person-view videos using a convolutional variational autoencoder and Gaussian processes

**DOI:** 10.3389/frobt.2022.903450

**Published:** 2022-09-30

**Authors:** Masatoshi Nagano, Tomoaki Nakamura, Takayuki Nagai, Daichi Mochihashi, Ichiro Kobayashi

**Affiliations:** ^1^ Department of Mechanical Engineering and Intelligent Systems, The University of Electro-Communications, Tokyo, Japan; ^2^ Department of Systems Science, Osaka University, Osaka, Japan; ^3^ Artificial Intelligence eXploration Research Center, The University of Electro-Communications, Tokyo, Japan; ^4^ Department of Statistical Inference and Mathematics, The Institute of Statistical Mathematics, Tokyo, Japan; ^5^ Department of Information Sciences, Ochanomizu University, Tokyo, Japan

**Keywords:** convolutional variational autoencoder, Gaussian process, hidden semi-Markov model, spatio-temporal categorization, segmentation, unsupervised learning

## Abstract

In this study, HcVGH, a method that learns spatio-temporal categories by segmenting first-person-view (FPV) videos captured by mobile robots, is proposed. Humans perceive continuous high-dimensional information by dividing and categorizing it into significant segments. This unsupervised segmentation capability is considered important for mobile robots to learn spatial knowledge. The proposed HcVGH combines a convolutional variational autoencoder (cVAE) with HVGH, a past method, which follows the hierarchical Dirichlet process-variational autoencoder-Gaussian process-hidden semi-Markov model comprising deep generative and statistical models. In the experiment, FPV videos of an agent were used in a simulated maze environment. FPV videos contain spatial information, and spatial knowledge can be learned by segmenting them. Using the FPV-video dataset, the segmentation performance of the proposed model was compared with previous models: HVGH and hierarchical recurrent state space model. The average segmentation F-measure achieved by HcVGH was 0.77; therefore, HcVGH outperformed the baseline methods. Furthermore, the experimental results showed that the parameters that represent the movability of the maze environment can be learned.

## 1 Introduction

Humans recognize continuous high-dimensional information by dividing and categorizing it into significant segments without explicit segmentation points. This unsupervised method has high generalizability and can be extended to mobile robots to help them adapt to various environments and contexts. Similarly, words or phonemes can be learned by segmenting speech, and unit motions can be learned by segmenting continuous motion data. Furthermore, spatio-temporal categories that are symbolized representations of a particular space can be learned via the segmentation of time-series visual information obtained as the agent moves around. This ability is also important for the spatial cognition of robots. Furthermore, it has been suggested that visual information contributes to spatial cognition in animals and humans. It has been reported that the brains of rats have place cells in their hippocampus (O’Keefe and Recce, 1993; Kitanishi et al., 2021) that are activated when the rat finds itself in a specific location and context; hence, the cells are considered to play an important role in navigation. It has also been reported that the hippocampus of bats plays an important role in the spatial navigation or recognition of their current position (Dotson and Yartsev, 2021). Moreover, Rolls and O’Mara (1995) and Rolls (1999) reported that view cells of a monkey, which are activated from visual information regardless of their location, affect spatial cognitive processing and help in spatial navigation. Spatial cognition is considered important not only in computational neuroscience but also in machine learning and robotics, and spatial cognition studies with robots have been conducted (Milford et al., 2004; Madl et al., 2015; Schapiro et al., 2017; Banino et al., 2018; Kowadlo et al., 2019; Scleidorovich et al., 2020). For advanced intelligent mobile robots, using information obtained by their own sensors to learn spatial knowledge is necessary (Taniguchi et al., 2018). Based on this background, this paper presents a stochastic model that divides and categorizes first-person-view (FPV) videos obtained by a mobile agent in a simulated maze. FPV videos contain spatial information, and the parameters representing the spatio-temporal structure can be learned by segmenting them.

**FIGURE 1:**
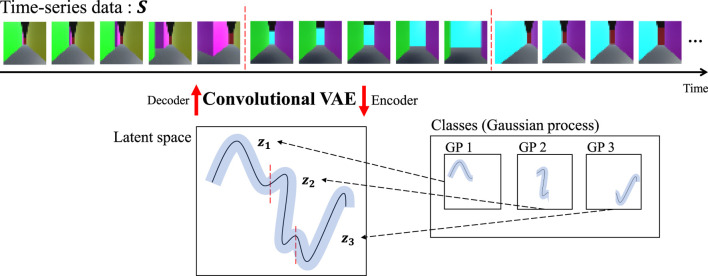
Generative process of the proposed method.

Previously, HVGH^1^ was proposed; it is an unsupervised segmentation method for time-series data that divides and classifies information using the hierarchical Dirichlet process-Gaussian process-hidden semi-Markov model (HDP-GP-HSMM). HVGH includes a variational autoencoder (VAE) that can be used as a feature extractor (Kingma and Welling, 2013). The parameters learned by the HDP-GP-HSMM (Nagano et al., 2018) are used as hyperparameters for the VAE, and parameters for HVGH are learned through the interaction between the VAE and the HDP-GP-HSMM process. It was confirmed that HVGH can estimate segments of motions more accurately than hidden Markov model (HMM)-based methods (Nagano et al., 2019). However, it was difficult for HVGH to segment videos in which significant features appeared among channels or pixels because HVGH extracts features using only fully connected layers. To overcome this limitation, a combined convolutional VAE (cVAE) and HVGH (HcVGH) are proposed; they enable feature extraction from videos while dividing and classifying them into significant segments. Furthermore, FPV videos obtained by a mobile agent in a simulated maze are used in this study. The images in such videos have spatio-temporal structure because the images temporally change under the spatial continuousness of the agent moving. Therefore, by segmenting such a video, HcVGH learns spatial categories and their transitions that represent both spatial and temporal changes. In this paper, we define such categorization as spatio-temporal categorization. Figure 1 presents an overview of the proposed model. Video data are compressed and converted into a latent variable sequence by the cVAE, and the latent variable sequence is divided and classified into segments using the same HDP-GP-HSMM process as before.

Herein, it is shown that the proposed method successfully segments and classifies variable sequences using a small number of video data. Following Kim et al. (2019), an experiment is conducted using FPV video data obtained by a mobile agent in a maze to demonstrate the superior stability and performance of HcVGH. By comparing HcVGH with HVGH, which uses only fully connected layers, it is demonstrated that capturing spatial characteristics in the image by convolution is effective for the estimation of the maze structure. Moreover, it is found that the proposed method has higher explainability regarding the spatial structure and segment classes than the end-to-end hierarchical recurrent state space model (HRSSM).

## 2 Related work

Several unsupervised time-series data changepoint detection methods, which assess fluctuation differences and repeated temporal similarities to identify potential change points, have been proposed (Yamanishi and Takeuchi, 2002; Lund et al., 2007; Liu et al., 2013; Haber et al., 2014). However, these methods do not necessarily indicate segment boundaries.

Many time-series data segmentation methods have been proposed (Lioutikov et al., 2015; Wächter and Asfour, 2015; Takano and Nakamura, 2016; Deldari et al., 2020). However, they make heuristic assumptions. For example, Wächter and Asfour (2015) proposed a method that uses contacts between an end-effector and an encountered object to segment object-manipulation motions. A method proposed by Lioutikov et al. (2015) requires segmentation candidate points in advance. Moreover, Takano and Nakamura (2016) proposed a method that leverages errors between predicted and actual values for robot observation segmentation. In another example, Deldari et al. (2020) developed an entropy and shape-aware time-series segmentation method that used segment similarities based on data mining without stochastic modeling. However, this method uses a threshold for computing segment length and similarity.

Notably, most proposed methods are probabilistically formulated using HMMs to segment time-series data (Beal et al., 2002; Fox et al., 2011; Taniguchi and Nagasaka, 2011; Matsubara et al., 2014). However, HMMs have difficulty handling complicated time-series patterns. By contrast, our model uses a GP, which nonparametrically represents complicated time-series data more appropriately than HMMs (Nakamura et al., 2017; Nagano et al., 2018).

Several studies have used deep neural networks to extract significant patterns from time-series data (Fraccaro et al., 2017; Rangapuram et al., 2018; Kurle et al., 2020). They proposed combination state-space models and deep neural networks to extract meaningful patterns; however, they focused not on segmentation but on prediction from observed time-series data considering their dynamics. By contrast, Liu et al. (2018) and Ansari et al. (2021) estimated the boundary points of segments and their classes by combining a recurrent neural network with a hidden semi-Markov model (Yu, 2010). In all cases, auxiliary variables are computed to represent the duration and boundaries of a state to segment time-series data. However, the number of classes is fixed, and only simple or periodic time-series data are used. Therefore, it is difficult for these methods to appropriately handle complex high-dimensional time-series video data acquired by a mobile robot.

Related to the proposed approach, high-dimensional time-series video data segmentation studies have been conducted (Kim et al., 2019; Tanwani et al., 2020). Tanwani et al. (2020) used labeled video data of robot-guided surgical operations to learn the latent space of suitable primitive motions. Semi-supervised learning for video segmentation was achieved by segmenting a video and relearning the latent space with a small number of manually labeled video segments. By contrast, the HRSSM was proposed to divide video data into primitive segments in an unsupervised, end-to-end manner using deep learning (Kim et al., 2019), and applied for a navigation task using divided segments in reinforcement learning. However, it is difficult for HRSSM to estimate classes. Furthermore, the method requires an inordinate amount of training data, and 1M frames of videos were used in the experiment. However, our proposed method can perform accurate segmentation using only about 1,000 frames. For application to real robots, the proposed method is considered more feasible from the viewpoint of cost for data collection than HRSSM.

Studies have investigated simultaneous mapping and categorization of the environment by mobile robots (Taniguchi et al., 2017; Chaplot et al., 2020). Chaplot et al. (2020) built a semantic map of object categories detected by pre-trained object detection and generated a path to the specified goal based on reinforcement learning. However, it required pre-trained object detection, and had difficulties in environments with objects whose categories could not be recognized by the pre-trained model or without characteristic objects. Taniguchi et al. (2017) proposed a method for a mobile robot to divide the space into place categories based on the position, visual features, and place-related utterances provided by the user. However, the place categories could not be learned without user utterances. Moreover, they used a pre-trained convolutional neural network to extract visual features, which is not a fully unsupervised method. By contrast, the aim of our proposed method is to segment space using only visual information and learn features extracted from images in an unsupervised manner.

## 3 Proposed Method

### 3.1 HcVGH

**FIGURE 2:**
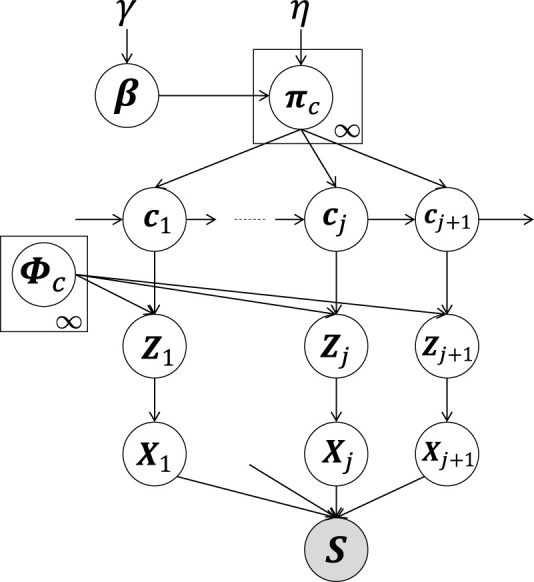
HcVGH model: White and gray nodes respectively represent unobserved variables and the high-dimensional observed sequence obtained by concatenating segments.

Figure 2 provides a graphical model of the proposed HcVGH, which is a generative model for segmenting time-series data. In HcVGH, it is assumed that the time-series data are generated based on the following process.

*β* represents an infinite-dimensional multinomial distribution and is generated by a Griffiths, Engen, and McCloskey (GEM) distribution (i.e., a stick-breaking process (Sethuraman, 1994; Pitman, 2002)) parameterized by *γ*. In Figure 2, *c*_*j*_ (*j* = 1, 2, … , *∞*) denotes segment classes. Moreover, ***π***_*c*_ represents the transition probability based on Dirichlet processes (Teh et al., 2006) parameterized by *η* and *β* as follows:

$$\beta ∼GEM\left(\gamma \right),$$ (1)

$$\pi_{c}∼DP\left(\eta ,\beta \right),$$ (2)

where *γ* and *η* represent the concentration parameters of the Dirichlet processes controlling the sparseness of the generated distribution. The two-phase Dirichlet process in Eqs 1, 2 is a hierarchical Dirichlet process (Teh et al., 2006).

In Figure 2, class *c*_*j*_ of the *j*th segment is generated by the (*j* − 1)th class, *c*_*j*−1_, and the transition probability, ***π***_*c*_. Moreover, latent variable ***Z***_*j*_ represents the *j*th segment generated by a GP (MacKay et al., 1998) based on the parameter, ***ϕ***_*c*_, corresponding to class *c* as follows:

$$c_{j}∼P\left(c|c_{j-1},\pi_{c}\right),$$ (3)

$$Z_{j}∼GP\left(Z|\phi_{c_{j}}\right).$$ (4)

Segments ***X***_*j*_ are generated from latent variables ***Z***_*j*_:

$$X_{j}∼p_{\mathrm{dec}}\left(X|Z_{j}\right).$$ (5)

Here, we assume that video data (i.e., a time series of images) comprise segment ***X***_*j*_. Hence, the cVAE’s decoder for *p*_*dec*_ is utilized to generate images ***X***_*j*_ from their low-dimensional latent variables, ***Z***_*j*_. The observation sequence, ***s*** = ***X***_1_, ***X***_2_, … , ***X***_*J*_, is obtained by combining ***X***_*j*_, based on these generative processes. Moreover, the sequence of latent variables, $\bar{s}=Z_{1},Z_{2},\ldots ,Z_{J}$ , is generated by connecting the segments of latent variables, ***Z***_*j*_ = ***z***_*j*1_, ***z***_*j*2_, … , ***z***_*ji*_, ⋯. Segment ***X***_*j*_ = ***x***_*j*1_, ***x***_*j*2_, … , ***x***_*ji*_, ⋯ is comprised of data points ***x***_*ji*_. The subscripts are omitted if the characters in the data point indicate the content.

The generative process of HcVGH is summarized in Algorithm 1, and the observed sequence, ***s***, is generated from this process. In the algorithm, the number of classes is assumed to be infinite, and there are infinitely possible class transitions. Notably, it is very difficult to directly implement this algorithm. To overcome this problem, a slice sampler (Van Gael et al., 2008) is used to produce a finite number of classes. In the slice sampler, an auxiliary variable, *u*_*j*_, is computed to truncate transitions with probability $\pi_{c_{j-1},c_{j}}<u_{j}$ .

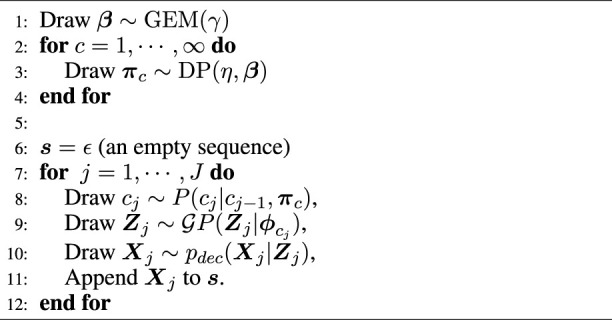

Algorithm 1 HcVGH ***s***-Generation Process

### 3.2 cVAE with GP prior

**FIGURE 3:**
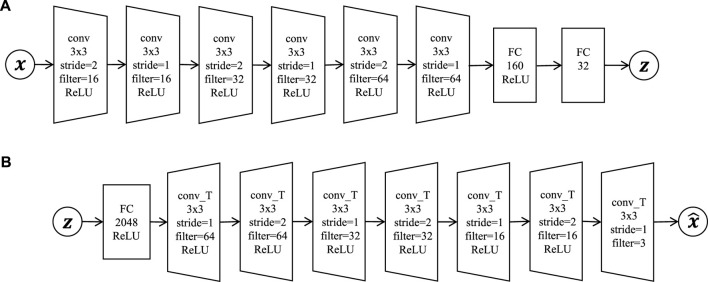
Convolutional variational autoencoder network architecture: **(A)** encoder = six convolutional layers (conv) and two fully connected layers (FC); **(B)** decoder = one fully connected layer and seven deconvolutional layers (conv_T).

To obtain a suitable latent variable, ***z***, of observation ***x***, a cVAE whose prior distribution is a GP was utilized. Figure 3A illustrates the encoder, and Figure 3B illustrates the decoder. In this figure, observed data point ***x*** is compressed into low-dimensional latent variable ***z*** through the encoder network, ***μ***_*enc*_(***x***), **Σ**_*enc*_(***x***):

$$z∼q_{\mathrm{enc}}\left(z\right)=N\left(z|\mu_{\mathrm{enc}}\left(x\right),\Sigma_{\mathrm{enc}}\left(x\right)\right),$$ (6)

where *q*_*enc*_(***z***) is a probability that approximates the posterior distribution, *p* (***z*** | ***x***). As a prior of ***z***, a Gaussian distribution with the mean vector, ***μ***_*c*_, and variance–covariance matrix **Σ**_*c*_ computed by $GP\left(z|\phi_{c}\right)$ is used:

$$p\left(z\right)=N\left(z|\mu_{c},\Sigma_{c}\right).$$ (7)

Using this prior, latent variables reflecting the characteristics of class *c* can be obtained. Moreover, the middle layer of the cVAE network is the convolution layer. Therefore, HcVGH efficiently compresses the time series of three-dimensional tensors (i.e., images, each of which is composed of height, width and channel).

The decoder network reconstructs observation $\hat{x}$ from latent variable ***z*** through the decoder network:

$$\hat{x}∼p_{\mathrm{dec}}\left(x|z\right).$$ (8)

### 3.3 Parameter inference

The log-likelihood of HcVGH is as follows:

$$\begin{array}{ll} \log p\left(X_{1},\ldots ,X_{J},c_{1},\ldots ,c_{J}\right) & =\log\underset{j}{\prod }\int_{Z_{j}}p\left(Z_{j},c_{j}\right)p\left(X_{j}|Z_{j}\right)dZ_{j}\\ & =\log\underset{j}{\prod }\int_{Z_{j}}\underset{\text{HDP}-\text{GP}-\text{HSMM}}{\underset{︸}{GP\left(Z_{j}|\phi_{c}\right)P\left(c_{j}|c_{j-1},\pi_{c}\right)}}\underset{\text{cVAE}}{\underset{︸}{p\left(X_{j}|Z_{j}\right)}}dZ_{j}. \end{array}$$ (9)

The factors, $GP\left(Z_{j}|\phi_{c}\right)P\left(c_{j}|c_{j-1},\pi_{c}\right)$ , are computed using HDP-GP-HSMM, and *p* (***X***_*j*_| ***Z***_*j*_) are computed with cVAE in Eq. 9. However, it is difficult to maximize Eq. 9 directly. To overcome this problem, the parameters are approximately maximized by alternately optimizing HDP-GP-HSMM and cVAE.

**FIGURE 4:**
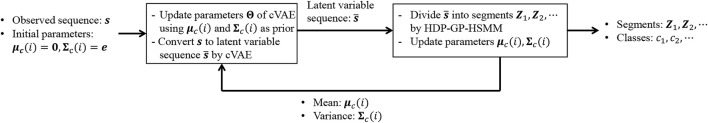
Overview of HcVGH parameter estimation. The parameters are learned using a mutual cVAE and HDP-GP-HSMM learning loop.

Figure 4 presents an overview of HcVGH’s parameter estimation process. First, the cVAE converts a sequence of observations, ***s*** = ***X***_1_, ***X***_2_, … , ***X***_*J*_, into a sequence of latent variables, $\bar{s}=Z_{1},Z_{2},\ldots ,Z_{J}$ . The cVAE parameters are estimated by maximizing the following variational lower bound:

$$L\left(x_{ji},z_{ji}\right)=\int q_{\mathrm{enc}}\left(z_{ji}|x_{ji}\right)\log p_{\mathrm{dec}}\left(x_{ji}|z_{ji}\right)dz_{ji}-wD_{KL}\left(q_{\mathrm{enc}}\left(z_{ji}|x_{ji}\right)\|p\left(z_{ji}|\mu_{c}\left(i\right),\Sigma_{c}\left(i\right)\right)\right),$$ (10)

where *w* represents the parameter used to weight the Kullback–Leibler divergence. If *w* > 1, it becomes possible to learn the disentangled latent variables that are suitable for segmentation (Higgins et al., 2017).

Then, the latent variable sequence, $\bar{s}$ , is divided and classified into segments ***Z***_1_, ***Z***_2_, … , ***Z***_*J*_ using HDP-GP-HSMM as follows:

$$\left(Z_{n,1},\ldots ,Z_{n,J_{n}}\right),\left(c_{n,1},\ldots ,c_{n,J_{n}}\right)∼p\left(\left(Z_{1},Z_{2},\ldots ,Z_{J}\right),\left(c_{1},c_{2},\ldots ,c_{J}\right)|{\bar{s}}_{n}\right),$$ (11)

where ***μ***_*c*_(*i*) and **Σ**_*c*_(*i*) are parameters of the predictive distribution computed by HDP-GP-HSMM and are used as parameters in the cVAE’s prior distribution in Eq. 10. Moreover, in the latent space learned by HcVGH, each latent variable reflects the characteristics of the time-series data and those of each class because the GP parameters differ for each.

In the proposed method, parameters of cVAE and HDP-GP-HSMM are optimized by repeating the above computations until the likelihoods converge.

As the proposed method is an improved version of HVGH, several detailed sections are omitted in this paper. Please refer to (Nagano et al., 2019).

## 4 Experiments

In this experiment, a small number of video data were divided and classified into significant segments using HcVGH to demonstrate that the estimated parameters express spatial structures. To evaluate the proposed HcVGH, it was applied to time-series data of FPV videos of an agent in a maze. For comparison, HRSSM (Kim et al., 2019) and HVGH (Nagano et al., 2019) were used as baselines.

### 4.1 Experimental setup

#### 4.1.1 Evaluation metrics

Four measures were used to evaluate segmentation accuracy: normalized Hamming distance, precision, recall, and F-measure. The normalized Hamming distance, an evaluation metric for clustering, ranges from zero to one, and a value closer to zero indicates approximation to ground truth. The remaining metrics range from zero to one as well, and larger values indicate that the estimated boundary points of segments are more similar to ground truth. With regard to the boundary point evaluation, it is very difficult to achieve a complete correspondence of an estimated boundary point and the ground truth; therefore, the estimated boundary was considered correct when it was within a tolerance of the ground truth. In this study, the tolerance was set to ±5% of the sequence length. Details of these metrics are described in (Nagano et al., 2019).

#### 4.1.2 Dataset

**FIGURE 5:**
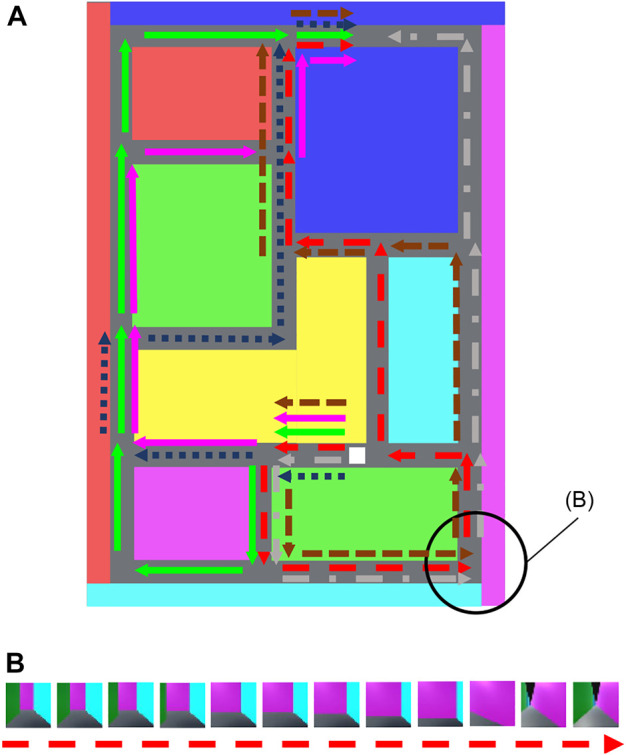
**(A)** Upper view of the maze. The white block indicates the agent, which can move along 6 paths indicated by the arrows. **(B)** Example of the first-person-view video data surrounded by the black circle in **(A)**.

Figure 5A presents the maze, and Figure 5B depicts FPV data of the agent in the maze. In this experiment, the agent moved along the 6 paths indicated by the arrows in Figure 5A. Each FPV data frame comprised red–green–blue image: $x_{i}\in R^{32\times 32\times 3}$ . To train HVGH, flattened vectors of the images were used. The maze consisted of 26 × 18 blocks, and the colored ones indicate areas where the agent could not traverse. In Figure 5A, the white block represents the agent, who can move “straight ahead,” “turn left,” and “turn right.” The agent moved straight ahead one block in five frames and rotated 90° to the left or right in three frames. The ground truth of the segment boundaries is the corners and T-junctions in the maze, and each hallway between boundaries is one segment.

To evaluate HRSSM, a 1M-frame dataset was constructed from the agent randomly selecting its action at the corner and T-junction with uniform distribution. The FPV data and maze used in this experiment are published at https://github.com/nagano28/color_maze.git.

#### 4.1.3 HRSSM

HRSSM requires many hyperparameters; it estimates whether there is a boundary in each subsequence, and 20 frames were set as the length of a subsequence. This length was used as the default value in the study. *L*_max_ is the maximum length of a segment, and *L*_max_ = 20 was set because a boundary may not exist in a subsequence. *N*_max_ is the maximum number of segments in a subsequence, and this parameter is influential to the segmentation. Therefore, HRSSM was trained by varying *N*_max_ = 1, 2, ⋯, 5. The number of dimensions of latent variables was set to 128, which was also set as the default value. For all other hyperparameters, the default values were used. With HRSSM, the paths indicated by the arrows in Figure 5 and the context of five frames of data before and after the path data frames were used. Furthermore, the learning iterations were repeated until the training loss converged.

HRSSM does not estimate classes but estimates the boundary points of the segments in an unsupervised manner. To evaluate the categorization capability of HRSSM, Gaussian mixture model was applied to the estimated latent variable and computed normalized Hamming distance. Moreover, to observe the influence of the training data size, HRSSM was evaluated in the following two cases.

• HRSSM (6 paths): training and testing on the 6-path dataset
• HRSSM (1M): training on the 1M dataset, testing on the 6-path dataset

#### 4.1.4 HcVGH

To compare HcVGH and HRSSM, the value of the required HcVGH parameter, *λ*, was changed to 20, 10, 7, 5, and 4, which corresponds to approximately *N*_max_ of HRSSM. *λ* is a mean parameter of the Poisson distribution, *P*_*len*_ (*k*|*λ*)^2^, that determines segment lengths. The parameters of the GP kernel function were the same as those used in the past work (Nagano et al., 2019). The weight of the regularization term of the cVAE was set to *w* = 5, and the number of dimensions of the latent variable was set to 16. When training the cVAE, 16 of the input data points were used as a mini-batch, and Adam (Kingma and Ba, 2014) was used for optimization with 100 iterations of updates. To train the HDP-GP-HSMM, the block Gibbs sampler was iterated eight times. Additionally, cVAE and HDP-GP-HSMM loops were repeated until the variational lower bound of the cVAE converged.

### 4.2 Results

#### 4.2.1 Segmentation results

Table 1 shows the results of segmentation using HRSSM, HVGH, and HcVGH. In this result, HRSSM’s estimation accuracy represents the most accurate result of learning iterations because in the preliminary experiment, we confirmed that the initial values did not significantly affect segmentation. In contrast, for HcVGH, we used the average value of the results of five executions with different initial values; this is because it has been empirically confirmed that the GP-HSMM-based model could sometimes not be able to get past the local optima.

**TABLE 1:** Baselines and HcVGH segmentation results.

	Hyperparameter	Hamming Distance	Precision	Recall	F-measure
HcVGH	λ = 20	0.33 ± 0.05	0.84 ± 0.06	0.91 ± 0.06	0.87 ± 0.06
	λ = 10	0.19 ± 0.02	0.68 ± 0.05	0.96 ± 0.01	0.79 ± 0.03
	λ = 7	0.18 ± 0.01	0.61 ± 0.03	1.0 ± 0.0	0.75 ± 0.02
	λ = 5	0.19 ± 0.01	0.56 ± 0.02	0.99 ± 0.01	0.72 ± 0.01
	λ = 4	0.19 ± 0.01	0.55 ± 0.02	1.0 ± 0.0	0.71 ± 0.02
	Average	0.22 ± 0.06	0.65 ± 0.12	0.97 ± 0.04	0.77 ± 0.07
HVGH	λ = 20	0.78 ± 0.18	0.54 ± 0.33	0.49 ± 0.35	0.50 ± 0.33
	λ = 10	0.66 ± 0.19	0.58 ± 0.33	0.56 ± 0.33	0.55 ± 0.32
	λ = 7	0.60 ± 0.26	0.34 ± 0.31	0.45 ± 0.42	0.39 ± 0.35
	λ = 5	0.68 ± 0.20	0.55 ± 0.34	0.55 ± 0.34	0.51 ± 0.29
	λ = 4	0.80 ± 0.21	0.20 ± 0.27	0.29 ± 0.39	0.23 ± 0.31
	Average	0.70 ± 0.20	0.44 ± 0.33	0.47 ± 0.35	0.43 ± 0.32
HRSSM (6 paths)	N_max_ = 1	0.40	0.95	0.56	0.70
	N_max_ = 2	0.41	0.72	0.23	0.34
	N_max_ = 3	0.41	0.79	1.0	0.88
	N_max_ = 4	0.40	0.62	0.96	0.76
	N_max_ = 5	0.40	0.39	1.0	0.55
	Average	0.40 ± 0.01	0.69 ± 0.21	0.76 ± 0.35	0.65 ± 0.21
HRSSM (1M)	N_max_ = 1	0.35	1.0	0.69	0.80
	N_max_ = 2	0.35	1.0	0.48	0.64
	N_max_ = 3	0.34	0.64	0.65	0.63
	N_max_ = 4	0.39	0.51	0.73	0.60
	N_max_ = 5	0.39	0.44	0.92	0.59
	Average	0.36 ± 0.03	0.72 ± 0.26	0.70 ± 0.16	0.65 ± 0.09

Table 1 shows that the F-measure of HcVGH and HRSSM (6 paths, *N*_max_ = 3) were both high, and the correct boundary points of the segments were estimated. However, this table also shows that the F-measure of HRSSM (6 paths) was strongly affected by the hyperparameter, *N*_max_. The normalized Hamming distance of HRSSM (1M) was shorter than that of HRSSM (6 paths). By contrast, regardless of the hyperparameter settings, the F-measure of HcVGH was stable, indicating high accuracy, and the Hamming distance of HcVGH was stably small. Finally, the accuracy of segmentation of HVGH was lower than that of the other methods.

From this result, it is considered difficult for HVGH whose VAE is composed of only fully connected layers to extract effective features to represent the maze. In HRSSM (6 paths), F-measure was not stable, and the maximum value was 0.88. This may be because the training dataset was too small for training HRSSM, and the parameters went into different local optima in each training. By contrast, the F-measure of HRSSM (1M) was stable although it was not higher than 0.88. The normalized Hamming distance of HRSSM (1M) became shorter than that of HRSSM (6 paths), and the latent variable capturing the characteristics of each category of FPV images could be learned through increasing the training data. However, in HcVGH, the average normalized Hamming distance was the smallest, the average F-measure was the highest, and their standard deviations were the smallest. This result shows that the dependence of HcVGH on a hyperparameter is less strong than that of HRSSM. The recall of HcVGH tended to be larger than its precision, and this was because it classified the corner and T-junction into a different category. Although this was judged as incorrect based on the definition of ground truth in this experiment, this estimation was also considered reasonable.

**FIGURE 6:**
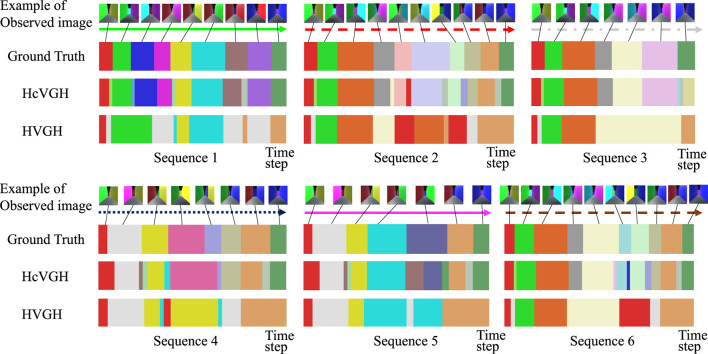
Segmentation results of a first-person-view video data.

Figure 6 shows the qualitative results of HcVGH and HVGH segmentation. In this figure, the horizontal axis represents the time step, and the color of the horizontal bar graph represents the class of the segments. The bar graph at the top indicates the boundary points and classes of the ground truth, and an example of an image observed by the agent is shown to correspond to the correct class. This figure demonstrates that the segments and classes estimated by HcVGH are approximate to the ground truth. However, the final segment of Sequence 3 was estimated to be a different class from the one obtained from the other sequences, even though the agents traversed the same corridor. This is because, depending on the direction of the agent, the image features were slightly different, even in the same corridor. Furthermore, some corners were classified as individual classes, which does not correspond with the ground truth. However, this estimation is reasonable and is not a problem. In HVGH, there were many misclassifications, and it can be seen that it is difficult to capture the characteristics of the images using only fully connected layers.

#### 4.2.2 Evaluation for spatial movability using transition probability

**FIGURE 7:**
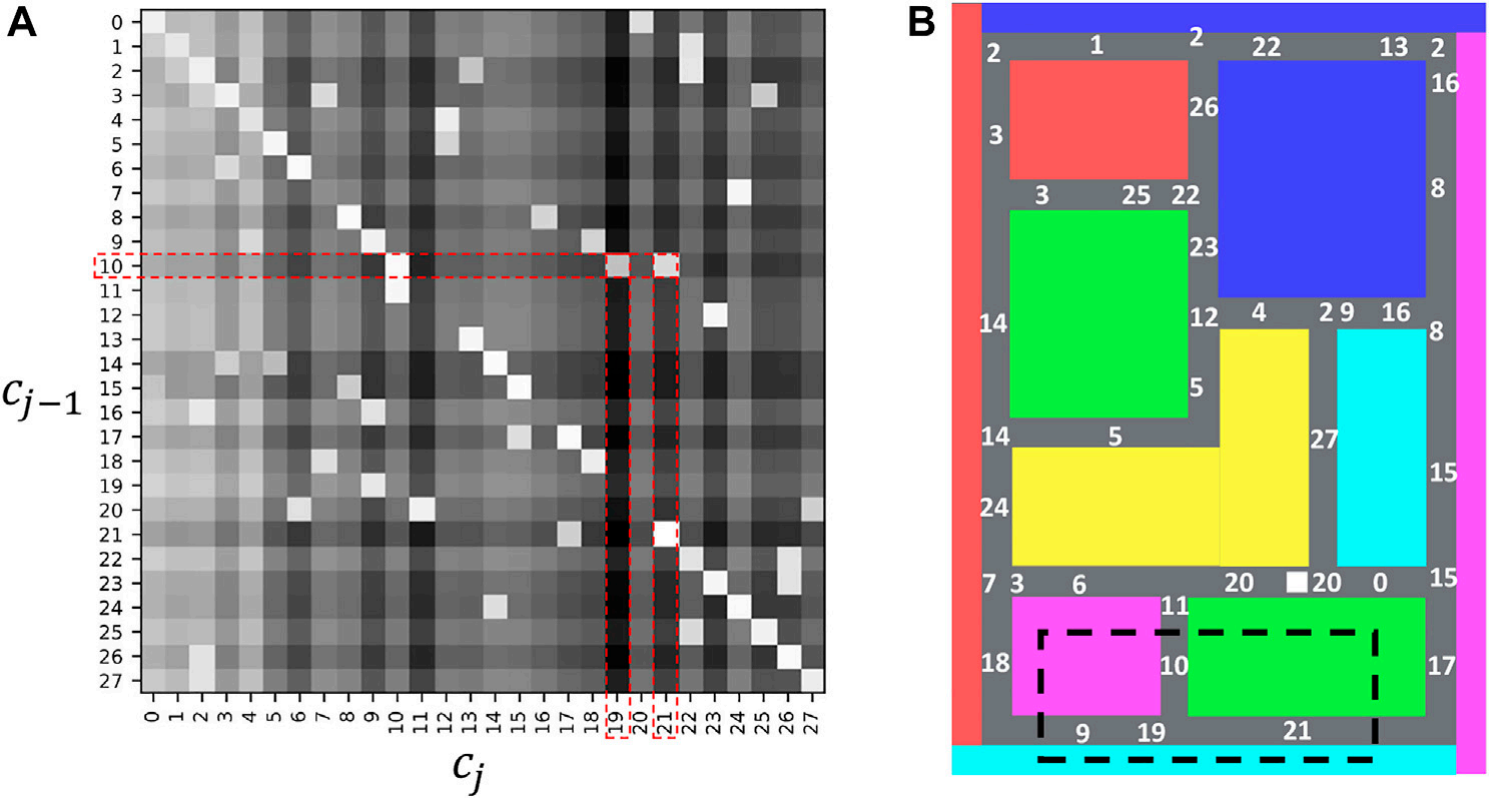
Transition probabilities of the estimated classes: **(A)** represents a transition matrix, and **(B)** represents the locations that correspond to each class in the maze.

Figure 7A presents a transition matrix that shows the transition probability of estimated classes. In this figure, the intensity of each cell represents log  *P* (*c*_*j*_|*c*_*j*−1_), and lighter values indicate higher probabilities. The white numbers in Figure 7B show the estimated classes, *c*, of HcVGH. As shown in Figure 7A, probabilities that represent movability from one place to another were explicitly obtained. For example, as seen in the red dashed rectangle of Figure 7A, the transition probabilities, log  *P* (*c*_*j*_ = 19|*c*_*j*−1_ = 10) and log  *P* (*c*_*j*_ = 21|*c*_*j*−1_ = 10), are high, and it was confirmed that they reflect actual transitions in the area enclosed by the black dashed line of Figure 7B.

Table 2 shows the class sequences that are paths in the maze and their normalized accumulated transition probabilities *L* computed as follows:

$$L=\frac{1}{J}\overset{J}{\underset{j=1}{\sum }}\log\hat{P}\left(c_{j}|c_{j-1}\right),$$ (12)

where $\hat{P}\left(c_{j}|c_{j-1}\right)$ is the transition probability without a prior distribution to prevent overestimation by GEM distribution, *J* is the length of the class sequence, and *L* is normalized by dividing by *J*. “T” in the “Type” column of the table shows the class sequences are included in the training data. “G” in the “Type” column of the table shows five paths with the highest probabilities in the randomly generated 100 paths by randomly dividing and connecting the class sequences of six paths in the training data. From this table, *L* of the six paths (paths 1–6) in the training data is higher. Furthermore, *L* of path 7, which circulates around the green right bottom block in Figure 7B, and paths 8 and 9, which combine paths from the training data, are also higher although the paths were not included in the training data. By contrast, *L* of paths 10 and 11, which contain spatially impossible transitions (underlined in Table 2), is lower. From this result, transition probabilities can represent spatial movability, and explicitly obtaining these probabilities is an advantage of HcVGH. However, spatial movability can sometimes be inaccurately estimated owing to misclassification. In our experiment, different corners were misclassified and estimated to belong to the same class; this caused the movability at these positions to be incorrectly estimated. To solve this problem, the number of misclassifications must be reduced.

**TABLE 2:** Evaluation of spatial movability of paths. “T” in the “Type” column shows their class sequences were included in the training data. “G” in the “Type” column shows generated class sequences that were not included in the training data. Underlined numbers in “class sequence” represent spatially impossible transitions.

	Type	Class sequence	*L*
1	T	20, 11, 10, 21, 17, 15, 8, 16, 9, 4, 12, 23, 26, 2, 22	−0.228
2	T	20, 6, 3, 7, 24, 14, 3, 25, 22, 26, 2, 22	−0.390
3	T	20, 6, 3, 7, 24, 14, 5, 12, 23, 26, 2, 22	−0.327
4	T	20, 11, 10, 21, 17, 15, 8, 16, 2, 13	−0.432
5	T	20, 11, 10, 21, 17, 15, 0, 20, 27, 2, 4, 12, 23, 26, 2, 22	−0.426
6	T	20, 11, 10, 19, 9, 18, 7, 24, 14, 3, 2, 1, 22	−0.531
7	G	20, 11, 10, 21, 17, 15, 0, 20, 11, 10, 21, 17, 15, 8, 16, 9, 4, 12, 23, 26, 2, 22	−0.244
8	G	20, 11, 10, 21, 17, 15, 0, 20, 11, 10, 19, 9, 18, 7, 24, 14, 5, 12, 23, 26, 2, 22	−0.297
9	G	20, 11, 10, 21, 17, 15, 0, 20, 11, 10, 21, 17, 15, 8, 16, 2, 13	−0.364
10	G	20, 11, 10, 21, 17, 15, 8, 16, 9, 4, 20, 11, 10, 21, 17, 15, 8, 16, 9, 4, 12, 23, 26, 2, 22	−4.397
11	G	20, 11, 10, 21, 17, 15, 0, 20, 11, 10, 20, 11, 10, 21, 17, 15, 8, 16, 9, 4, 12, 23, 26, 2, 22	−4.420

**FIGURE 8:**
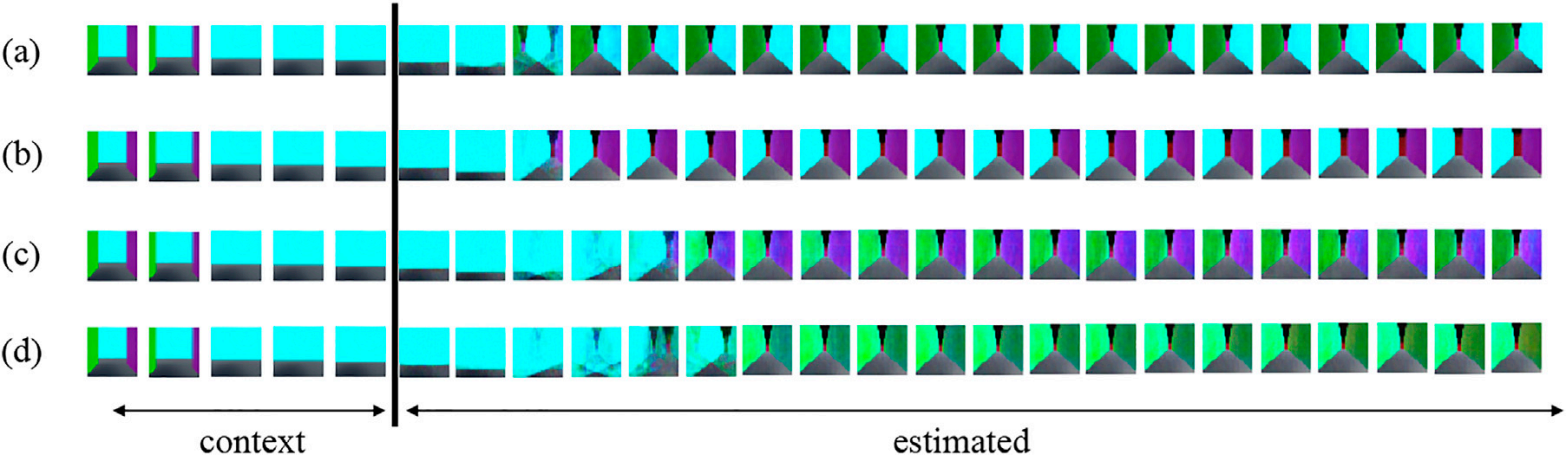
Estimated results by HRSSM: **(A–D)** respectively represent an example of predicted images by using the same contexts.

However, in HRSSM, subsequent states are generated from the current state by a recurrent neural network, and it is difficult to explicitly obtain movability between states. To evaluate movability estimated by HRSSM, the number of transitions at T-junction in the blacked dashed rectangle in Figure 7B of predicted 100 paths by the most accurate model HRSSM (1M, *N*_max_ = 1) were manually counted. Figure 8 shows examples of prediction and Table 3 shows the counting result. Figure 8A shows the agent prediction that it is possible to turn left, and Figure 8B shows the agent prediction that it is possible to turn right. The agent actions are determined by uniform distribution in the 1M dataset; however, the prediction was biased as shown in Table 3. Moreover, HRSSM predicted images that mixed both the left- and the right-side paths (Figure 8C) and mixed the left/right-side and other paths (Figure 8D). From this result, we find that in HRSSM, rough transition probabilities can be computed manually or by any other additional means, but they cannot be obtained explicitly. Therefore, it is difficult to evaluate spatial movability with HRSSM in an unsupervised manner.

**TABLE 3:** Number of the predicted paths at the T-junction.

	left	right	else
HRSSM (1M)	52	23	25

#### 4.2.3 Comparison of latent variables

**FIGURE 9:**
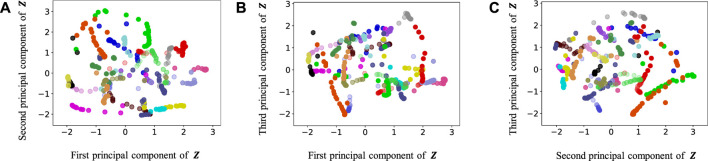
Latent variables learned by HcVGH: **(A–C)** respectively represent the first and second, first and third, and second and third dimension of the principal component of the latent variables. The color of each point reflects the correct corridor class.

**FIGURE 10:**
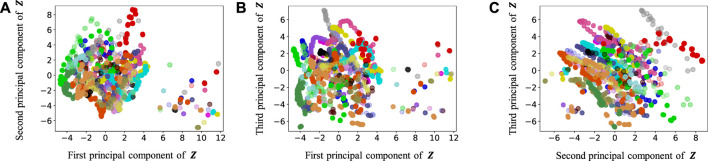
Latent variables learned by HRSSM (6 paths): **(A–C)** respectively represent the first and second, first and third, and second and third dimension of the principal component of the latent variables. The color of each point reflects the correct corridor class.

**FIGURE 11:**
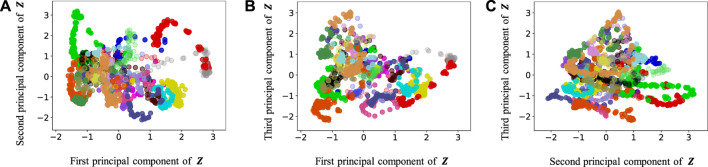
Latent variables learned by HRSSM (1M): **(A–C)** respectively represent the first and second, first and third, and second and third dimension of the principal component of the latent variables. The color of each point reflects the correct corridor class.

**FIGURE 12:**
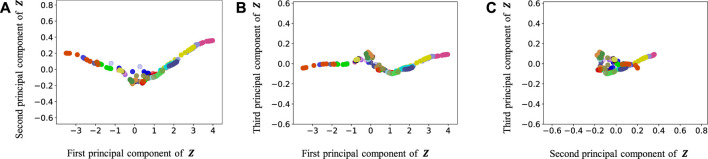
Latent variables learned by HVGH: **(A–C)** respectively represent the first and second, first and third, and second and third dimension of the principal component of the latent variables. The color of each point reflects the correct corridor class.

**FIGURE 13:**
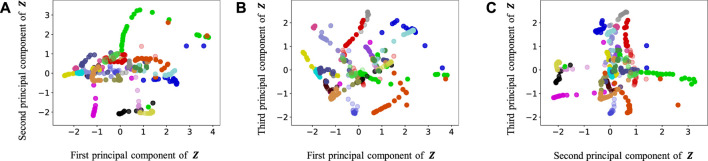
Latent variables learned by only cVAE: **(A–C)** respectively represent the first and second, first and third, and second and third dimension of the principal component of the latent variables. The color of each point reflects the correct corridor class.

Figures 9–13 show the latent variables of 6 paths estimated by HcVGH, HRSSM (6 paths), HRSSM (1M), HVGH, and only cVAE. In these figures, panels (a), (b), and (c) respectively represent the first and second, first and third, and second and third dimensions of the latent variables, which were compressed via principal component analysis (Pearson, 1901). The color of each point reflects the correct corridor class.

In Figure 12, the latent variable of HVGH is not well separated for each class. Similarly, in Figure 10, the latent variables of HRSSM (6 paths) are not separated for each class, and compared with HRSSM (6 paths), the latent variables of HRSSM (1M) seem to improve slightly in Figure 11. However, the latent variables of HRSSM (6 paths) and HRSSM (1M) are overlapped and their separation for each class is inadequate for clustering them. Therefore, it is difficult to classify the latent variables into place categories in an unsupervised manner.

By contrast, in HcVGH (Figure 9), the latent variables of the same class have more similar values, and the latent variables of different classes are well separated. Compared with those of only cVAE (Figure 13), the latent variables of HcVGH (Figure 9) are better separated. This is because *μ* and *σ* computed by HDP-GP-HSMM are used as the prior of cVAE in the HcVGH, and therefore, the latent variables that are classified into the same categories get closer.

### 4.3 Discussion

From the results, it can be seen that HcVGH is accurate and stable regardless of the hyperparameters. By contrast, HRSSM tends to be affected by hyperparameters, and parameter tuning is required depending on the training data.

Furthermore, HcVGH has high explainability because the transition probabilities that are considered movabilities can be obtained explicitly. These transition probabilities can be used for global path planning, and various paths can be planned according to a purpose. For instance, shortest path, longest path, paths going through a particular location, or paths that maximize a particular objective function can be planned considering the movability. However, HRSSM does not have such explicit parameters, and therefore, it is difficult to plan paths according to a purpose. One solution is generating many paths and selecting one path that matches the purpose; however, the optimal path is not always generated. Another solution is computing transition probabilities as in Section 4.2.2; however, generated samples require manual classification. For this reason, HcVGH is suitable for application to mobile robots.

However, HcVGH has limitations. It depends on visual information only, and locations with similar appearances can be misclassified. To overcome this limitation, integration of the method that can deal with multimodal information such as a joint multimodal VAE (Suzuki et al., 2016) should be considered. Moreover, by integrating the slam-based method such as (Taniguchi et al., 2017), the robot can learn the place concept in a fully unsupervised manner avoiding misclassification.

Another limitation of the model is its scalability. The size of the dataset used for HcVGH training was not very large because the proposed method uses a Gaussian process whose computational cost to train *N* data is *O*(*N* ^3^). Therefore, it would be difficult to apply this to a huge dataset. In the future, a verification of the scalability of the proposed method will be conducted by using realistic huge data such as car-camera videos (Geiger et al., 2013).

## 5 Conclusion

In this article, a cVAE was integrated into HVGH, a model developed in a past work, and HcVGH, which divides and classifies video time-series data into segments, was proposed. The experimental results show that HcVGH achieved more accurate FPV video data segmentation than the baseline methods. Moreover, the results showed that HcVGH has high explainability and a high segmentation accuracy when compared with HRSSM, which segments video data in an end-to-end manner. HcVGH estimates boundary points and classes of segments more stably than HRSSM.

Furthermore, in HcVGH, the parameters that represent spatio-temporal structure of the maze can be obtained explicitly. Using these parameters, spatial movability can be evaluated, which is useful for navigation planning. In the future, the agent’s actions will be introduced, and a method to plan its actions based on probabilistic inference (Levine, 2018) using HcVGH will be formulated.

However, one of the limitations of HcVGH is the misclassification caused by using unimodal information. In the future, the cVAE of HcVGH will be extended to a joint multimodal VAE to divide and classify multimodal information to overcome this problem. Another limitation of HcVGH is its scalability. Therefore, it will be necessary to verify the scalability of HcVGH by performing segmentation on a larger dataset, and more realistic dataset.

## Data Availability

The datasets presented in this study can be found in online repositories. The names of the repository/repositories and accession number(s) can be found in the article/Supplementary Material.

## Footnotes

1 HVGH: Hierarchical Dirichlet process-Variational autoencoder-Gaussian process-Hidden semi-Markov model

2

$P_{\mathrm{len}}\left(k|\lambda \right)=\frac{\lambda^{k}e^{-\lambda }}{k!}$
